# Benthic biogeographic patterns in the southern Australian deep sea: Do historical museum records accord with recent systematic, but spatially limited, survey data?

**DOI:** 10.1002/ece3.4565

**Published:** 2018-11-08

**Authors:** Jason E. Tanner, Franziska Althaus, Shirley J. Sorokin, Alan Williams

**Affiliations:** ^1^ South Australian Research and Development Institute (Aquatic Sciences) Adelaide South Australia Australia; ^2^ CSIRO Ocean and Atmosphere, Marine Laboratories Hobart Tasmania Australia; ^3^ Present address: Centre for Marine Bioproducts Development Flinders University Bedford Park SA 5042 Australia

**Keywords:** continental slope, deep sea, demersal fish, epifauna, marine megafaunal invertebrates, southern Australia

## Abstract

**Aim:**

To document biogeographic patterns in the deepwater benthic epifauna and demersal fishes of southern Australia, and determine whether museum records and systematic survey data provide matching results.

**Location:**

Southern Australian (32–44^o^S) continental slope (200–3,000 m deep).

**Taxon:**

Marine benthic fauna (Arthropoda, Bryozoa, Cnidaria, Echinodermata, Mollusca, Porifera, Sipuncula, and fishes).

**Methods:**

All available electronic records of fauna from the above taxa and ≥200 m depth off the southern Australian coastline, regardless of organism size, were collated from Australian museums and checked for geographic and taxonomic consistency. These records were then split into 40 geographic segments of roughly equal numbers, with each segment then treated as a sample in multivariate analyses of assemblage composition. Data from a recent (2015) systematic beam trawl survey along five north–south transects in the central Great Australian Bight were also included for comparison.

**Main conclusions:**

The systematic survey data grouped with the associated geographic segments despite differences in sampling technique (single gear compared to multiple gears), with subsequent differences in taxonomic biases, and the use of a 25 mm mesh, which would undersample some smaller organisms present in the museum data. Thus, the museum data and the survey data provided the same results for the central Great Australian Bight at the level of the whole assemblage. The main biogeographic break occurred off southeastern Tasmania, with a second substantial break occurring at around the border between New South Wales and Victoria. This indicates the potential for unused museum data to describe biogeographic patterns over regional spatial scales, especially in the deep sea where the expense of collecting new data is relatively high.

## 1 INTRODUCTION

Biogeographic affiliations in the deep sea are often poorly understood (e.g., Linse, Griffiths, Barnes, & Clarke, 2006; Sutton et al., 2017; Watling, Guinotte, Clark, & Smith, 2013), due to the difficulty and expense in collecting comprehensive data on the assemblages present in these areas. For example, experience shows that fewer than six trawl samples of benthic fishes and epifaunal invertebrates can be taken in 3,000 m water depth in a day, simply due to time necessary for the gear to be safely deployed to the bottom and hauled back up. Considerable time, money, and expertise are required to build a broad‐ranging and quality‐assured collection of faunal data. The question then arises, can the collective records held by museums for an area of interest provide an alternative source of biogeographic data to new field collections?

Museum historical data will typically be represented by records from multiple collections, using a wide variety of sampling techniques, and made over a long period of time, typically many decades or longer. However, these records often result from ad hoc sample collection, or surveys that documented only certain components of fauna. Where historical catch data are available, they may not be comprehensive, as many surveys only targeted taxa of interest (e.g., fishes), or had too few resources to undertake finely resolved taxonomic identifications. Even when records stem from a comprehensive survey effort, the associated metadata are often not archived in a way that can be matched to individual specimen records, and/or the comprehensive survey data are simply no longer available, especially for earlier surveys where they only ever existed as written records. In addition, many samples collected well before the advent of modern navigational technology have limited or inaccurate location and depth data. Finally, many specimens have not been subject to rigorous taxonomic scrutiny, or identifications may have been based on outdated taxonomy. As a consequence of these potential limitations, it is not clear how useful even a large collection of historical records is for assessing biogeographic patterns in assemblage composition without substantial further work to verify taxonomy. While such records have been used successfully for this purpose in the past, it has generally been reliant on the time‐consuming development of a carefully curated and verified database of samples for an individual taxonomic group, usually a single class or phylum (e.g., Clark et al., 2010; Griffiths, Arango, Munilla, & McInnes, 2011; Linse et al., 2006; O'Hara, Rowden, & Bax, 2011; Woolley et al., 2016).

Shallow coastal waters, in contrast, are much more well known (e.g., Costello & Chaudhary, 2017), and therefore, biogeographic patterns in shallow waters are often better understood than those in the deep sea (e.g., Colgan, 2016), as they are more accessible and thus have been the focus of much more intensive sampling. However, as the physical environment, and subsequently the life histories of the organisms living there, can differ substantially between the shallow and deep oceans, it is not possible to extrapolate shallow‐water biogeographic patterns to the deep. Biogeography and patterns in diversity are known to differ with depth (Macpherson et al., 2010; Williams, Althaus, Clark, & Gowlett‐Holmes, 2011; Woolley et al., 2016), and it is generally considered that deep‐sea species are widely distributed compared to their shallow‐water counterparts (Gooday & Jorissen, 2012; McClain & Hardy, 2010). However, recent work on benthic and particle‐attached microbes suggests that many “species” in the deep sea have a relatively restricted distribution, leading to substantial geographic structuring in assemblages (Bienhold, Zinger, Boetius, & Ramette, 2016; Salazar et al., 2016; Zinger et al., 2011). An interest in the biogeography of deep‐sea benthic fauna in the Great Australian Bight (GAB) off southern Australia has been recently stimulated by proposed oil and gas industry development. As part of a study of the region's benthic ecosystems, we wanted to evaluate the potential of the collective historical record to generate a biogeography of the southern Australian deep‐sea (200–3,000 m depth) benthic epifauna and fishes based on available electronic specimen records held by major Australian museums. We had the opportunity to determine how well this historical data defined the assemblages present by comparing it to a series of systematic beam trawl surveys using a 25 mm mesh that collected benthic invertebrate epifauna and demersal fishes undertaken during our study in the central GAB in late 2015 (Williams, et al., 2018a, 2018b). This is a particularly data‐poor region, with records from our field program almost doubling the number of records from the entire south‐facing Australian slope, thus providing a challenging test of the ability to describe biogeographic affiliations using historical data.

To date, there have been few studies published that provide a detailed quantitative examination of the biogeography of Australian marine waters, including three on fishes (Commonwealth of Australia, 2006; Last et al., 2005; Lyne et al., 2009), one on ophiuroids (O'Hara, 2008), one on sponges (Hooper & Ekins, 2004), and one on algae (Waters et al., 2010), although the Australian region is also considered in a number of larger scale analyses (e.g., O'Hara et al., 2011; Woolley et al., 2016). Only Last et al. (2010) and O'Hara (2008) consider deep (>200 m) waters off southern Australia. The first five of these studies involved comprehensive collation of records from a wide range of museums, along with published literature, followed by re‐examination of many specimens. For each individual taxon considered, taxon distributions were then modeled based on the available point data, interpolating species occurrence between these points, before the final biogeographic analysis was undertaken. While this approach provides a gold standard, it is also very labor‐intensive and therefore takes years to complete. We were interested in determining if similar results could be obtained from historical museum data using a much less exhaustive validation and analysis process. A similar approach to ours is taken by Waters et al. (2010) for southern Australian algae, although they use the presence/absence in bioregions to test whether previously defined provinces exist for this group, rather than attempting to produce a biogeographic map de novo.

## 2 METHODS

Database extracts were requested from museums throughout Australia for all specimen lots with collection depth ≥200 m from temperate Australia (collection location between 32°S and 44°S). Data were received from the Australian Museum (AM), Museums Victoria (MV), Queensland Museum (QM), Western Australian Museum (WAM), and the Northern Territory Museum of Arts and Sciences (NTM). Although the South Australian Museum (SAMA) had substantial holdings of relevant specimens, very few had been databased. Consequently, a systematic search of the museum shelves was undertaken, and all marine specimen records from ≥200 m depth were databased, with the exception of arthropods, of which only the decapods and pycnogonids were entered due to resource constraints (although other arthropod taxa are present in the other data sets included in the analysis). Large deepwater collections, particularly from waters east of Tasmania, are held by the Tasmanian Museum and Art Gallery, but it was not possible to extract individual sample data from their system. Due to time constraints, and to more closely match this data set to the taxa included in the beam trawl data set detailed below, analysis was restricted to the eight major taxa listed in Table 1.

**Table 1:** Data for electronic data holdings of deepwater (≥200 m) temperate invertebrates (seven selected phyla) and fishes at Australian museums and used to examine biogeographic patterns in southern Australia's deepwater marine fauna

Phylum	AM	MV	NTM	QM	SAMA	WAM	Total records	Total species
Arthropoda	2,385	3,500	9	12	364	43	6,313	792
Bryozoa	80	11		1	68		160	52
Cnidaria	225	114	38	18	351	4	750	91
Echinodermata	1,090	1,266			394	46	2,796	265
Mollusca	5,083	1,202	7		127	226	6,645	681
Porifera	35	6	3	126	93	17	280	15
Sipuncula	22	4			7		33	3
Fishes	3,343			7			3,350	538
Total	12,263	6,103	57	164	1,404	336	20,327	2,437

Note

Total species only includes those taxa identified to species level. AM: Australian Museum; MV: Museums Victoria; NTM: Northern Territory Museum; QM: Queensland Museum; SAMA: South Australian Museum; WAM: Western Australian Museum. Note that Arthropoda in the SAMA data only is restricted to decapods and pycnogonids.

The collated data set was subject to screening and taxonomic updating as follows. Nineteen hundred and sixty‐six records that were identified in the original museum records as having a spatial precision >10 km were discarded, along with 188 that had no spatial position, and 174 that were identified as being >4,000 m depth. The remaining records were then mapped in ArcGIS (v10.3.1 ESRI Inc.), along with a comprehensive bathymetry of Australia, an integrated product previously produced at CSIRO (G. Keith), based on the Geoscience Australia (GA) GA2009 250 m bathymetric product (Whiteway, 2009) refined with bathymetric data based upon LiDAR/LADS surveys and surveys based on acoustic systems provided by CSIRO and GA, and historical soundings provided by the Australian Hydrographic Service (AHO) and the Western Australian Department of Primary Industries and Regional Development (WA DPIRD). Those records that mapped on land (412), in water shallower than 180 m (1,260) or in water >3,000 m (430) were also discarded, along with three records that mapped well outside the southern Australian continental slope (one each from Africa, the Australian North‐West Shelf, and the Chatham Rise). While it is likely that a number of records still had unidentified positional errors, these would degrade our ability to detect any spatial patterns, rather than produce spurious patterns, and no attempt was made to go back to original survey reports or other documentation to try and verify the positional data in the remaining records.

The remaining 20,327 records were then cross‐checked against the World Register of Marine Species (WoRMS) by calling the web service from within Microsoft Excel to automatically update the names of all taxa to improve taxonomic consistency by eliminating recorded synonymies. Specimens that had not been identified to species level were not re‐examined, but were instead retained at the taxonomic level in the original museum records. All tentative species designations (sp 1, cf sp y, sp z? etc) were moved up to genus level. Species that could not be found in WoRMS were carefully checked for possible mis‐spellings, and if no species name could be confirmed, also moved up to the genus level.

**Figure 1:**
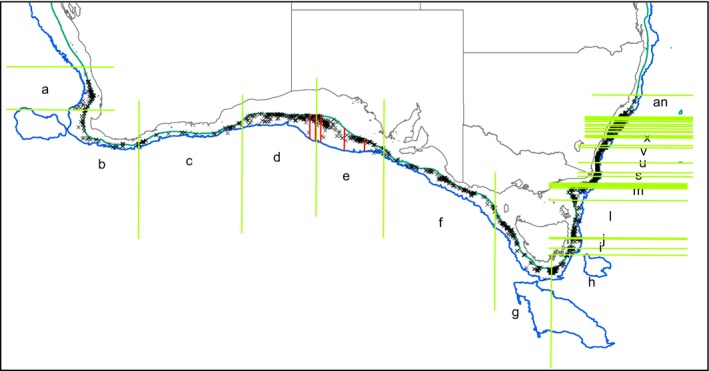
Map of southern Australia showing geographic distribution of samples represented in museum collections (black crosses), with geographic segments used for multivariate analysis (indicated by green vertical/horizontal lines and letters—missing letters indicate that the segment is too small to label). Inner and outer bathymetry contours are 200 and 3,000 m, respectively. Red vertical lines in segments d and e indicate location of the GAB benthic transects (T1 to west, T5 to east)

For analysis, records were grouped by splitting the region into 40 contiguous geographic segments around the coast, each segment with ~508 records (Figure 1). This often split records that appeared to be from a single station between two adjacent segments. In this case, the split point was moved so that all records from a single station were included in a single segment (so the range in sample size was 368–533). While this approach resulted in 33 segments on the east coast of Australia, which were often very small when assessed by coastline length or seabed area, and seven much larger segments spread over the much more extensive south and west coasts, it was taken to avoid analytical issues that would result from very low sample sizes in some segments if an equal area approach was taken. Split points were not constrained to coincide with previously suggested biogeographic boundaries, as we were aiming to generate a biogeography de novo, rather than to test previous biogeographies. Consequently, all statistical analyses are exploratory rather than confirmatory.

To determine whether fauna collected during a recent systematic beam trawl survey of the Great Australian Bight would show consistent distributional patterns with the museum records detailed above, we combined and compared the data sets. Beam trawl data were collected as part of the Great Australian Bight Research Program (GABRP) collected from the Marine National Facility RV *Investigator* in December 2015 (IN2015_C02) (described in more detail in Williams, et al., 2018a, 2018b). Briefly, beam trawls were conducted at predetermined depths (200, 400, 1,000, 1,500, 2,000, and 3,000 m) along each of five north–south transects in the central GAB (see Figure 1). All fauna caught in each trawl were sorted, classified, and enumerated on retrieval, and representative samples were kept for further detailed taxonomy by specialist taxonomists. For the purpose of the analysis presented here, the detailed taxonomy is utilized, and each transect is treated as a single depth‐integrated sample, with the data from stations at 200, 400, 1,000, 1,500, 2,000, and 3,000 m being pooled (as the museum data are depth‐integrated). Again, we have restricted the analysis to the groups listed in Table 1. Although the beam trawl had a 25 mm mesh size and therefore did not generally retain smaller organisms, no attempt was made to screen museum records by size class, as this information was generally not available.

To assess spatial patterns in deep‐sea assemblage structure, we used non‐metric multidimensional scaling (nMDS), and cluster analysis using group average linkage and a similarity profiles analysis based on 999 permutations to indicate significant groupings at the 5% level. All analyses were performed on the presence/absence data, as the museum data are unlikely to provide a good indication of abundance. This also removes issues associated with the potential for duplicate samples to have been lodged in separate museums. Bray–Curtis dissimilarities (equivalent to the Sorenson index when applied to the presence/absence data) were used so that joint absences did not inflate the apparent similarity between segments, as neither the museum nor the survey data sets can be considered to provide a comprehensive list of taxa present, and the absence of a species in the data set cannot be considered absence from the sample region. To determine whether patterns were consistent between different taxonomic levels, we repeated the analyses at the species, genus, family, class, and phylum levels. In each case, specimens that had not been classified down to the level being analyzed were retained in the analysis at the taxonomic level recorded. A two‐stage nMDS was then used to assess concordance between the patterns at each level. Separate analyses were also conducted for each phylum and again compared using a two‐stage nMDS. Multivariate analyses were undertaken in Primer (v7.0.11, Primer‐E Ltd.).

## 3 RESULTS

The final museum “species” list contained 3,496 taxa (at the lowest taxonomic level recorded). Of these, 1,248 were represented by a single record, although one record may have equated to multiple individuals. A total of 2,993 taxa (85.6%) were represented by 10 or fewer records. A total of 25 taxa had 50 or more records, although three of these were undifferentiated groupings only identified to the family, order, or class level. In total, 2,437 taxa were identified to species level (Table 1).

The survey data set contains 1,853 records from 617 taxa, 264 with only a single record. Of these 617 taxa, only 359 are present in the museum data set. The two most abundant species in the museum data set (and six of the 10 most abundant) were absent from the survey data set, these being *Sassia kampyla* (Gastropoda, 233 museum records), *Fusitriton retiolus* (Gastropoda, 97), *Rexea solandri* (silver gemfish, 96), *Columbarium hedleyi* (Gastropoda, 92), *Nassarius ephamillus* (Gastropoda, 89), and *Fusinus annae* (Gastropoda, 72). The four most common species in the museum data set shared with the survey data set were *Mallavium devotum* (Gastropoda, 96 museum records, 1 survey specimen), *Fissidentalium ponderi* (Scaphopoda, 77, 4), *Semicassis pyrum* (Gastropoda, 76, 1), and *Columbarium pagodoides* (Gastropoda, 71, 4). Eight of the ten most common species in the survey data set were represented in the museum data set *Parapagurus richeri* (Decapoda, 18 survey specimens, 2 museum records), *Nematocarcinus productus* (Decapoda, 16, 3), *Ophiomusa lymani* (Ophiuroidea, 15, 24), *Halosauropsis macrochir* (Actinopterygii, 13, 3), *Coryphaenoides filicauda* (Actinopterygii, 12, 0), *Ebalia tuberculosa* (Decapoda, 10, 25), *Ophiomusia scalare* (Ophiuroidea, 10, 20), *Brucerolis victoriensis* (Isopoda, 10, 10), *Synaphobranchus brevidorsalis* (Actinopterygii, 10, 0), and *Amphiophiura urbana* (Ophiuroidea, 9, 24). The full list of taxa included in both data sets is provided in Supporting Information Table S1.

**Figure 2:**
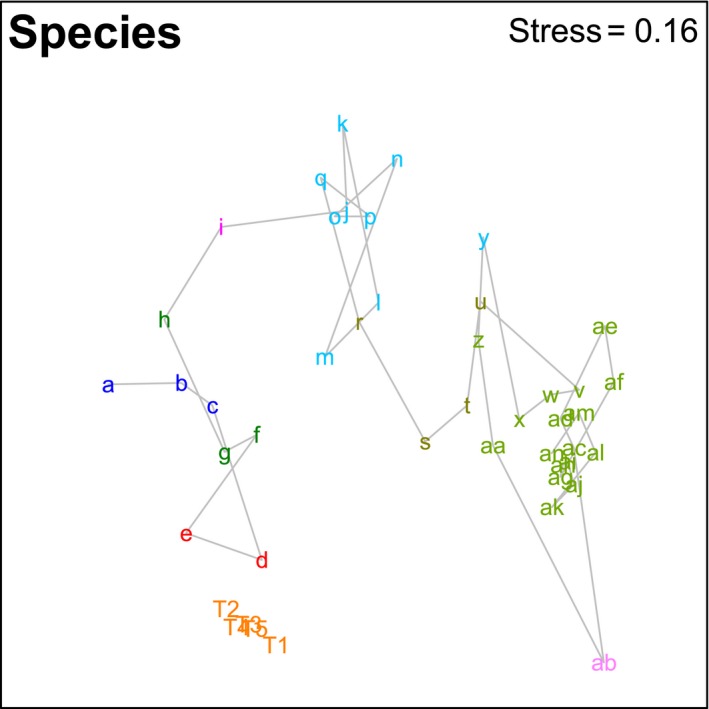
Non‐metric multidimensional scaling ordination plot showing species‐level biogeographic patterns in deep‐sea benthic assemblages around southern Australia (see Figure 1 for geographic locations of each point). The line connects geographically contiguous segments from west (a) to east (an). Color coding indicates 20% similarity level from the cluster analysis in Figure 3

**Figure 3:**
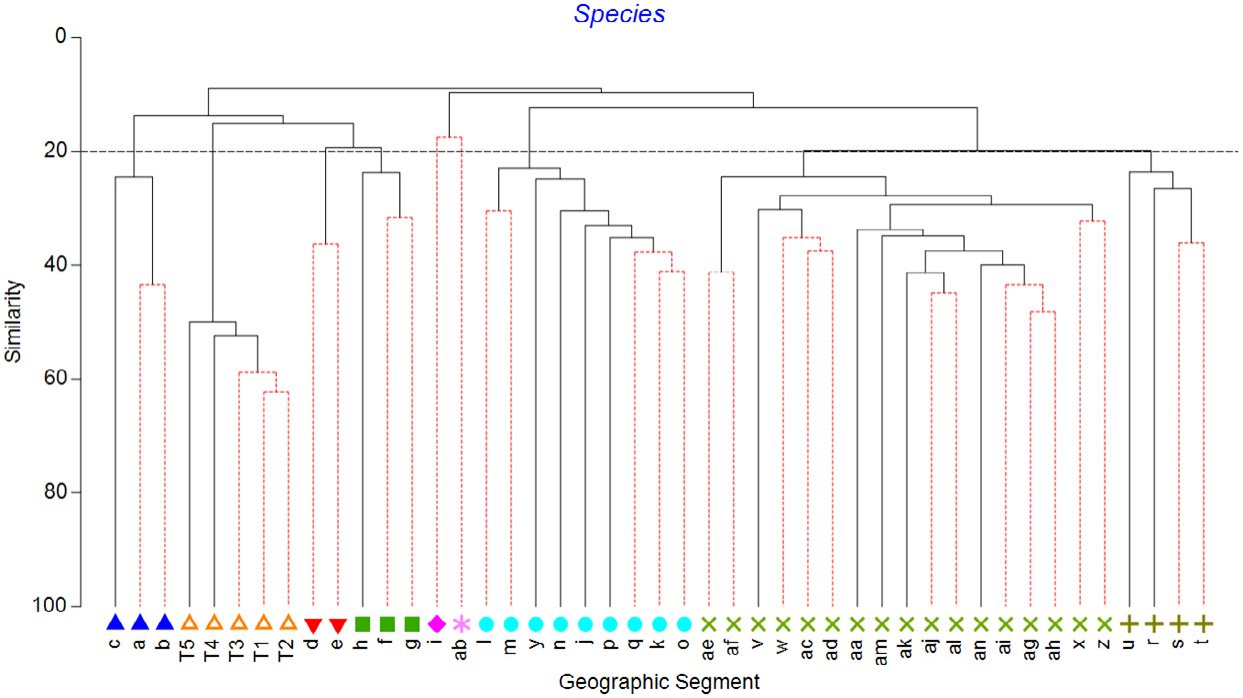
Cluster analysis of southern Australian deep‐sea benthos at the species level. Symbols and colors indicate groupings at the 20% similarity level used in Figure 2. Red lines indicate groupings that do not differ at the 5% significance level according to similarity profiles analysis

**Figure 4:**
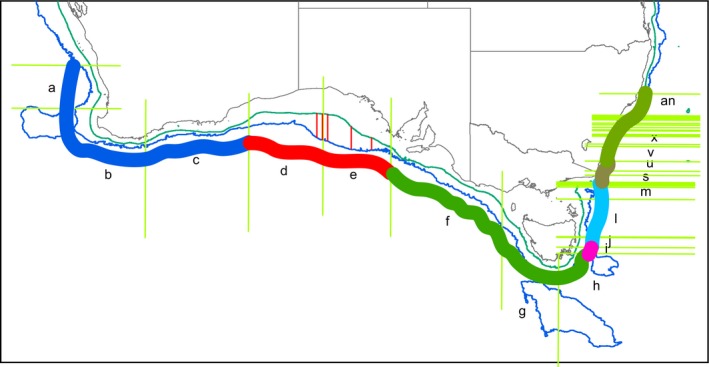
Map of southern Australia showing biogeographic zones (thick multicolored line around the coast, coding matching Figures 2 and 3) based on the multivariate analyses at the species level. Zone boundaries are based on a similarity cutoff of 20% in the cluster analysis (Figure 3). Individual geographic segments used for multivariate analysis are indicated by green vertical/horizontal lines and letters (missing letters indicate that the segment is too small to label). Inner and outer bathymetry contours are 200 and 3,000 m, respectively. Red vertical lines in segments d and e indicate location of the GAB benthic transects (T1 to west, T5 to east). Note that segment ab is different to the surrounding segments, but is too small to appear on the map

**Figure 5:**
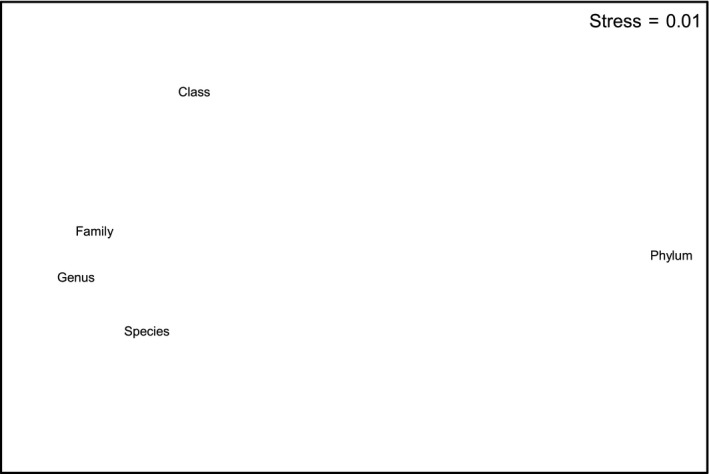
Results of the second‐stage nMDS showing degree of concordance in biogeographic patterns at different taxonomic levels. Points that are close together indicate that the analyses at the respective taxonomic levels (i.e., the plots in Supporting Information Figure S1) show very similar patterns, while those that are more distant do not show similar patterns

There were clear geographic patterns present in the museum data, with the ordination showing the southwest and south coasts grouping with southeast Tasmania, a second grouping covering northeast Tasmania through to about the border between Victoria and New South Wales, and a third grouping of New South Wales sites (Figure 2). This is supported by the cluster analysis, which also splits off the southwest segments from the southern segments and shows more detailed structuring along the east coast (Figures 3 and 4). The survey data form a tight grouping in both the ordination and the cluster analysis, lying very close to geographic segments d and e, within which they lie. These patterns persist at the genus and family levels, start to break down at the class level, and are entirely absent at the phylum level (Supporting Information Figures S1 and S2). The concordance of patterns at the species, genus, and family levels is confirmed by the second‐stage nMDS (Figure 5), which shows these three levels all lying close together in the ordination, with class and phylum lying in substantially different parts of the plot. While the similarity profiles analysis (Figure 3) suggests that there are 28 different clusters in the data set at the 5% significance level, this does not provide a useful regional‐scale biogeography, and so for the purposes of discussing broader biogeographic patterns, we have chosen to plot eight regions in Figure 4 based on the 20% similarity level in the cluster analysis.

“Species”‐level patterns do not appear to be driven by geographic patterns in taxa not identified to species level, as all taxa with a correlation >0.8 with the nMDS axes are identified to species level (Supporting Information Figure S1). However, at the genus level, a number of the taxa with high correlations are only identified to the family level or higher. The grouping of segments off the New South Wales coast is characterized by gastropods, particularly those in the genus *Columbarium* and the family Olividae. Those of northeast Tasmania and Victoria are characterized by the presence of a range of isopod taxa, including the families Dendrotionidae, Janiridae, and Munnidae, as well as the cumacean family Gynodiastylidae. Southern and southwestern segments are typified by Sipuncula, anemones in the family Hormathiidae, sponges of the families Suberitidae and Leucaltidae, ophiuroids in the genera *Amphiophiura* and *Ophiomusia*, and octocorals of the genus *Umbellula*.

**Figure 6:**
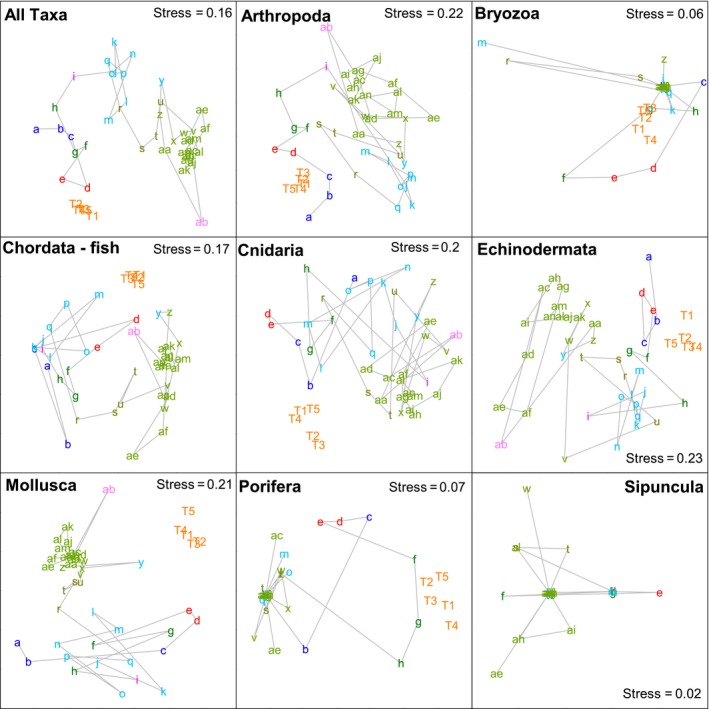
Non‐metric multidimensional scaling ordination plots showing biogeographic patterns in individual phyla around southern Australia (see Figure 1 for geographic locations of each point). Color coding indicates 20% similarity level from the cluster analysis in Figure 3

**Figure 7:**
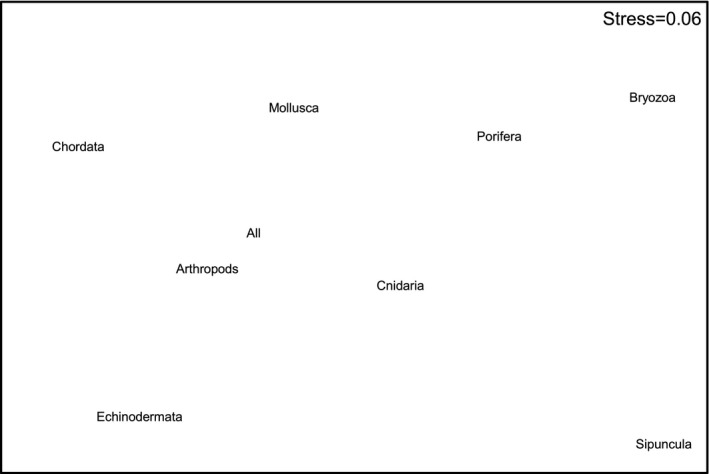
Results of the second‐stage nMDS showing degree of concordance in biogeographic patterns in different phyla. Points that are close together indicate that the analyses at the respective taxonomic levels (i.e., the plots in Figure 6) show very similar patterns, while those that are more distant do not show similar patterns

When individual phyla were analyzed, only the Arthropoda, which had the second largest sample size, produced similar results to the all taxa analysis (Figures 6 and 7). Similar groupings could also be seen in the ordination for Echinodermata (Figure 6), although the second‐stage ordination suggests that this pattern is less similar to the all taxa pattern than those produced for Chordata, Cnidaria, and Mollusca. The Porifera, Bryozoa, and Sipuncula produced the most dissimilar results, although these three phyla all had very low sample sizes (Table 1).

## 4 DISCUSSION

There was a strong pattern of geographic grouping in the historical museum data and a clear concordance between it and the newly collected beam trawl survey data. The new samples grouped with the spatially conterminous geographic segments in both the ordinations and the cluster analyses at the species, genus, and family levels. Although less obvious, this pattern persisted at the class and even phylum level, despite the analyses being based on the presence/absence of only eight phyla. Our results demonstrate that existing data for marine benthic fauna have the potential to identify biogeographic patterns over large distances, in continental slope depths at least. Museum collections, individually or collectively, contain large volumes of relevant information that has potential for analysis once compiled and quality‐assured, but the utility of such data may be overlooked. In instances where time is limited, or financial or taxonomic resources are unavailable for new collections, an analysis of historical data may be a feasible option for examining biogeographic patterns over a regional spatial scale. This potential may be relatively strong in the deep sea where the expense of collecting new data is relatively high and taxonomy for many higher level taxa relatively poorly known.

The museum data showed assemblages present in the central GAB (d, e in the figures) were most closely related to those further east on the southern Australian continental slope (f, g, h) and then to the western GAB (c) and southwest corner of Australia (a, b). Assemblages off southeastern Tasmania (i) appeared to be intermediate between southern Australia and the southeast (j‐r), although the cluster analysis indicates that the transition between h and I is the biggest break point. There is another transition around the border between Victoria and New South Wales (NSW) (r‐u), with the remainder of the NSW coast forming a distinct and fairly tight group. The one exception was segment ab, which was an outlier in the species‐, genus‐, and family‐level analyses. This segment had the lowest number of records (368, compared to the next lowest being 441), possibly indicating that this is insufficient to properly characterize it. The transition between h and i appears to correspond to the bifurcation between the eastward‐flowing Zeehan Current and the south‐flowing East Australian Current (EAC) (Middleton and Cirano, 2005). The former would promote dispersal of the southern Australian fauna around to the southeast of Tasmania, while the later would act as a barrier to dispersal further up the Tasmanian coast. The EAC would promote strong connectivity between northeast Tasmania and the east coast of mainland Australia. With the strength of the EAC increasing in recent times, it is possible that this eastern Australian fauna will move further south, as has already been well documented in shallower waters (e.g., Ling, Johnson, Ridgway, Hobday, & Haddon, 2009). The differences between the fauna off northeast Tasmania and Victoria on the one hand, and NSW on the other, may relate to the Bass Strait Cascade, which results in an outflow of warm salty water down the slope in this region (Middleton & Black, 1994), and which may impede dispersal of deeper fauna southwards.

Although there were clear geographic groupings in the benthic assemblages, the overall level of similarity between different geographic segments was relatively low (often only 30%–40%), even when they were located close together. This is likely to be heavily influenced by the sample size in each segment only being 10%–15% of the total number of taxa included in the analysis. Larger sample sizes, however, would quickly result in southern Australia being subsumed into a single segment, providing no differentiation in the region from which the new survey data were collected. Alternatively, the number of taxa could be reduced by decreasing the spatial extent of the analysis (e.g., by excluding data from New South Wales), although this in turn would reduce the ability to put southern Australian assemblages into a broader temperate Australian context. Interpolating taxon ranges on the assumption that taxa not recorded from segments between the extremes of their range are present and just not sampled would also help to increase the similarity of adjacent segments, but makes the analysis process much more laborious and risks recording a taxon as present when it is genuinely absent.

There have been two previous studies of biogeographic patterns in southern Australian deep‐sea fauna, one for fishes (Last et al., 2005) and the other for ophiuroids (O'Hara, 2008). The patterns found here are broadly in agreement with these two single‐taxon analyses, although with some differences. Both indicate a southern Australian province that extends further west than our central GAB province, incorporating segment c, and for fishes some of segment b, and then a southwest zone in segment b, with segment a being part of a larger central western province. To the east, the southern province, and for fishes a transition zone, includes segment f and some of g. There is then a Tasmanian province roughly corresponding to segments g and h (and into i for fishes). The east coast of Tasmania and the NSW coasts then form two additional provinces. Both of these previous studies used point source data to model the distribution of each species considered and put considerable effort into examining specimens to ensure consistent taxonomy. In contrast, we were interested in determining if museum data were sufficiently robust to determine broad‐scale biogeographic patterns without this extra work—although it should be noted that our analyses benefited from updated taxonomies of fishes and ophiuroids that resulted from the previous studies. There are of course many other advantages to going back to the original museum specimens, which is still required to develop a detailed understanding of the distribution and ecology of specific taxa, and to fully understand the assemblages present in specific regions.

Our patterns are also broadly consistent with the global biogeographic provinces proposed for the lower bathyal (800–3,500 m) by Watling et al. (2013) based purely on modeled environmental variables (temperature salinity, dissolved oxygen, and particulate organic carbon flux). They propose an Indian province that extends across western and southern Australia, a sub‐Antarctic province that includes southern Tasmania, and a New Zealand‐Kermadec province that includes temperate and subtropical eastern Australia. In intertidal and shallow subtidal waters, there are well‐documented phylogeographic breaks off eastern Victoria, southern NSW, southeastern Tasmania, and between the central and eastern GAB (Colgan, 2016; Teske, Sandoval‐Castillo, Waters, & Beheregaray, 2017). Interestingly, these all appear to coincide with biogeographic breaks found here, including the two major breaks off southeastern Tasmania and eastern Victoria.

A clearly obvious pattern in the museum data is the vast preponderance of records from the eastern coast of Australia compared to the south and southwest. Of the 20,327 records used in total, <3,000 were from the extensive southern coastline, while >16,000 were from the east coast. Particularly, well‐sampled areas lie off the central east coast of Victoria and the NSW coast between Jervis Bay and Sydney. The additional 1,853 records from the beam trawl survey thus represent almost half of the data available from southern Australia, although only covering a very small proportion of this region. This east–west imbalance in the historical record overemphasizes the affinities of our invertebrate megafauna and fish collections (Williams, et al., 2018a) with the western Pacific Ocean compared to the Indian Ocean. Another consequence of the limited sampling off southern Australia is that the spatial resolution in this region is very low, and the boundaries between different biogeographic provinces may not be well demarcated. This lack of resolution may also account for the identification of a single large southwestern province, compared to several smaller provinces in previous biogeographic analyses based on single taxonomic groups (Last et al., 2005; O'Hara, 2008). There is clearly a need for a substantial increase in survey effort in the southern and southwestern Australian region if we are to properly understand what fauna are present, let alone any ecological patterns that they exhibit. This bias is likely to be related to Australia's major population centers, and hence largest museums and other marine research organizations, and its longest standing fisheries, all lying on the east coast.

We have not considered the potential for different depth biomes to be present in our analyses, primarily due to the rapid decline in number of records as depth increases making sample sizes potentially inadequate along the southern Australian coastline. Biogeographic provinces along southern Australia, which are based on demersal fishes, differ substantially between the shelf (<200 m deep) and upper slope (200–1,200 m) (Commonwealth of Australia, 2006), and our beam trawl collections show patterns of fish distributions vary with depth (more cosmopolitan species and many fewer endemics in continental slope/rise compared to shallower depths (Williams, et al., 2018b)). However, while there were substantial differences in taxa present in different depth biomes (50–1,500 m depth), O'Hara (2008) found broadly consistent biogeographic patterns for ophiuroids. Similar results have been found for galatheid squat lobsters in the Pacific Ocean, with factors structuring biogeography apparently the same on the continental slope (200–900 m depth) and continental rise (>900 m) (Macpherson et al., 2010). In contrast, at a global scale, there are substantial differences in diversity patterns in ophiuroids above and below 2,000 m depth (Woolley et al., 2016).

Classification of a fauna into biogeographic provinces depends very much on the scale of the study. For example, although O'Hara (2008) identifies a number of provinces in southern Australia, based on Australian data only, O'Hara et al. (2011) identify only two depth‐dependent strata when undertaking an analysis across the broader Australasian region, with the bathyal province being shared with New Zealand. Similarly, a global analysis of seamount fishes groups southeastern Australia with New Zealand (Clark et al., 2010).

At the phylum level, the Arthropoda produced the most similar patterns to those seen in the all taxa analysis. While this may be partly explained by sample size, there were more mollusk records in the data set than arthropods. Each of these phyla had approximately double the number of records as the next two most common (Echinodermata and Chordata [fishes]), although both of these groups were as good as the mollusks at replicating the overall pattern. Even the Cnidaria, with 8–9 times fewer records than the Mollusca and Arthropoda, replicated the overall pattern as effectively as the Mollusca. Thus, rather than being an artifact of sample size, it appears that the differences between phyla may be real differences in biogeographic structure, although this needs to be confirmed by more detailed analysis of these phyla along the lines of those that have been conducted for fishes (Last et al., 2005) and ophiuroids (O'Hara, 2008). The lack of congruence in the biogeographic patterns in the Porifera, Bryozoa, and Sipuncula is likely to be related to low sample size, with all of these taxa accounting for <1.5% of the total number of records used. As different taxa, including groups within phyla, can differ in their life‐history strategies, trophic ecology, substrate requirements, and other factors, it is not axiomatic that all will show similar biogeographic patterns. For example, O'Hara and Poore (2000) showed some differences between echinoderms and decapods in southern Australia, and Piacenza et al. (2015) have shown different patterns of diversity between taxa on the United States west coast. Here, although the Porifera and Bryozoa had low sample size, they group somewhat with the Cnidaria on the right of Figure 7. All three phyla are composed predominantly of benthic suspension feeders. Conversely, Echinodermata, Arthropoda, and Chordata (fishes) lie toward the left of this figure and are all predominantly mobile, while Mollusca with a mix of sedentary suspension feeders and more mobile species lies in between. Thus, these similarities and differences in ecological characteristics could be influencing the degree of concordance in the biogeographic patterns.

While the museum and survey data sets produce similar assemblages when viewed in the context of the overall variation seen across southern Australia, there are still some substantial differences in the taxa included. Only two thirds of taxa documented in the survey are included in the museum data, and the majority of the most abundant species in the museum data are absent from the survey. In part, this is due to the sparsity of museum data from the southern coast of Australia, meaning that east coast species that are absent from the south coast are over‐represented. It is also notable that eight of the 10 most abundant species in the museum data were gastropod mollusks, with a ninth being a scaphopod mollusk. This may indicate a sampling bias in the museum data, with mollusks over‐represented as they are of interest to a greater range of people (shell collectors), and more taxonomically amenable than many other groups, as well as being more likely to be collected intact from a deepwater trawl. The museum data are also likely to be based on a wider range of collection techniques and may thus capture species that are not amenable to sampling using a beam trawl.

Overall, we found a very close correspondence between the assemblages documented in our beam trawl surveys in the central GAB and the conterminous assemblages documented from museum records. There are also strong similarities between the biogeographic groupings found here and those found in previous detailed studies of fishes and ophiuroids, and the limited systematic survey data available for analysis also suggest that the patterns found are real. Based on the results presented here, the central deep GAB appears to show some differences to adjacent areas in the GAB, but has affinities with the fauna found in the eastern GAB and around southwestern Australia. There is a very clear distinction between the faunas in the south and southwest, and those present along the east coast.

## AUTHOR CONTRIBUTIONS

JT, SS, and AW conceived the study; JT undertook the analysis and led the writing; SS and FA collated the museum data and maintained the database; AW, JT, and SS undertook new sample collection; and all authors contributed to the preparation of the paper.

## DATA ACCESSIBILITY

Data from the 2015 survey will be made available via the CSIRO data trawler (https://www.cmar.csiro.au/data/trawler) in June 2018. Historical museum data are the property of the respective museums and can be accessed by request to these institutions.
