# Supplementary material for: Benthic biogeographic patterns in the southern Australian deep sea: Do historical museum records accord with recent systematic, but spatially limited, survey data?
Source: Ecol Evol. 2018 Nov 8;8(23):11423–33. doi: 10.1002/ece3.4565 (PMC6303719; doi:10.1002/ece3.4565)
Supplement: Supplementary file 1 [file ECE3-8-11423-s001.pdf]

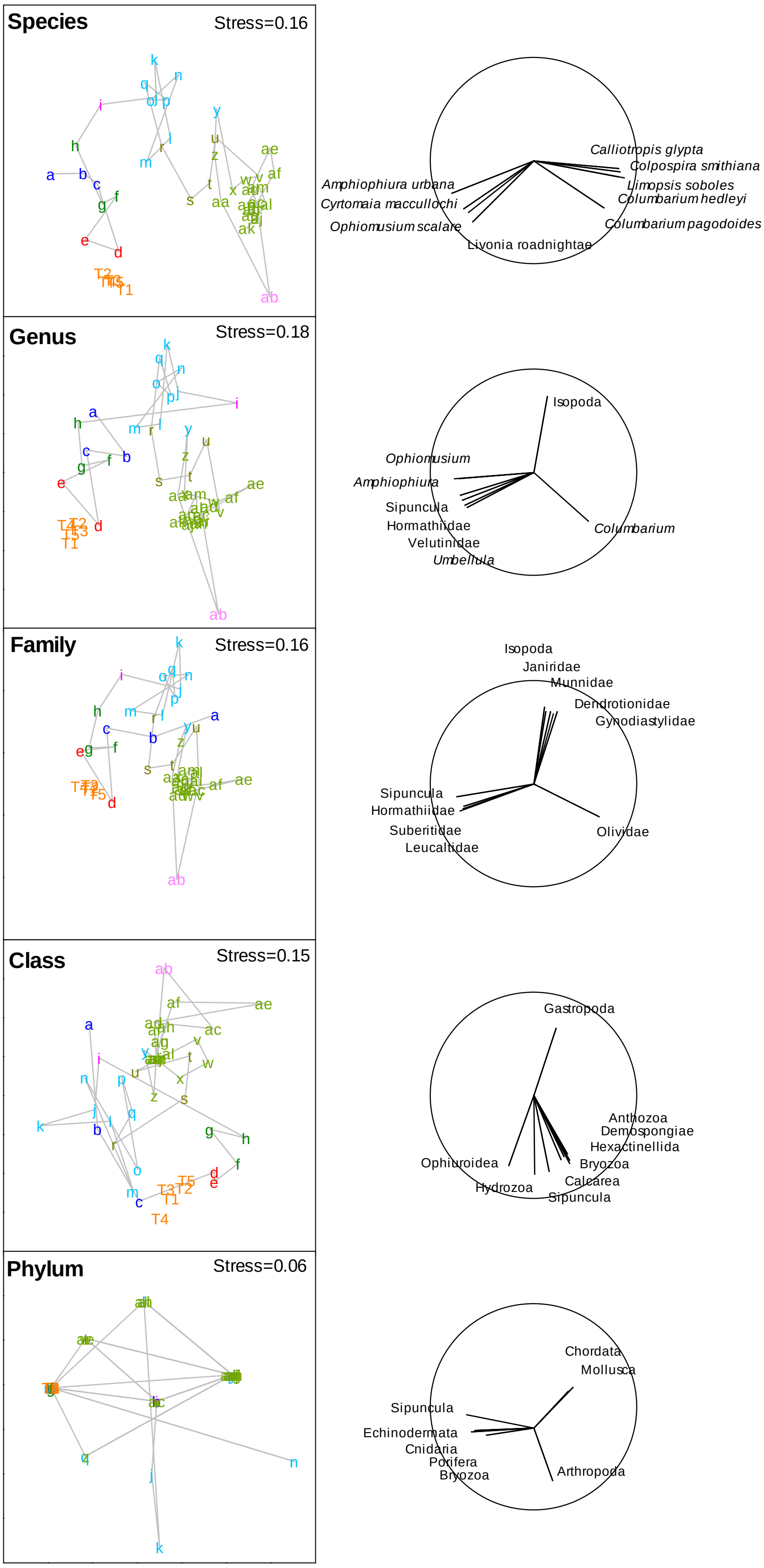

Species
Stress=0.16
Calliotropis glypta
Colpospira smithiana
Amphiophiura urbana
Limopsis soboles
Cyrtomaia macculochi
Columbarium hedleyi
Ophiomusium scalare
Columbarium pagodoides
Livonia roadnightae
Genus
Stress=0.18
Isopoda
Ophiomusium
Amphiophiura
Sipuncula
Hormathiidae
Columbarium
Velutinidae
Umbellula
Family
Stress=0.16
Isopoda
Janiridae
Munnidae
Dendrotionidae
Gynodiastylidae
Sipuncula
Hormathiidae
Suberitidae
Olividae
Leucaltidae
Class
Stress=0.15
Gastropoda
Anthozoa
Demospongiae
Hexactinellida
Ophiuroidea
Bryozoa
Hydrozoa
Calcarea
Sipuncula
Phylum
Stress=0.06
Chordata
Mollusca
Sipuncula
Echinodermata
Cnidaria
Porifera
Bryozoa
Arthropoda

Supplementary Figure 1 Nonmetric Multidimensional Scaling ordination plots showing biogeographic patterns in deep-sea benthic assemblages off southern Australia (see Figure 1 for geographic locations of each point). Biplots indicate taxa most highly correlated to the nMDS axes (using Pearson correlations), with the circle representing a correlation of 1. Colour coding indicates 20% similarity level from the species level cluster analysis in Figure 3. Note that the species-level plot is the same as in Figure 2, and is presented again for ease of comparison.

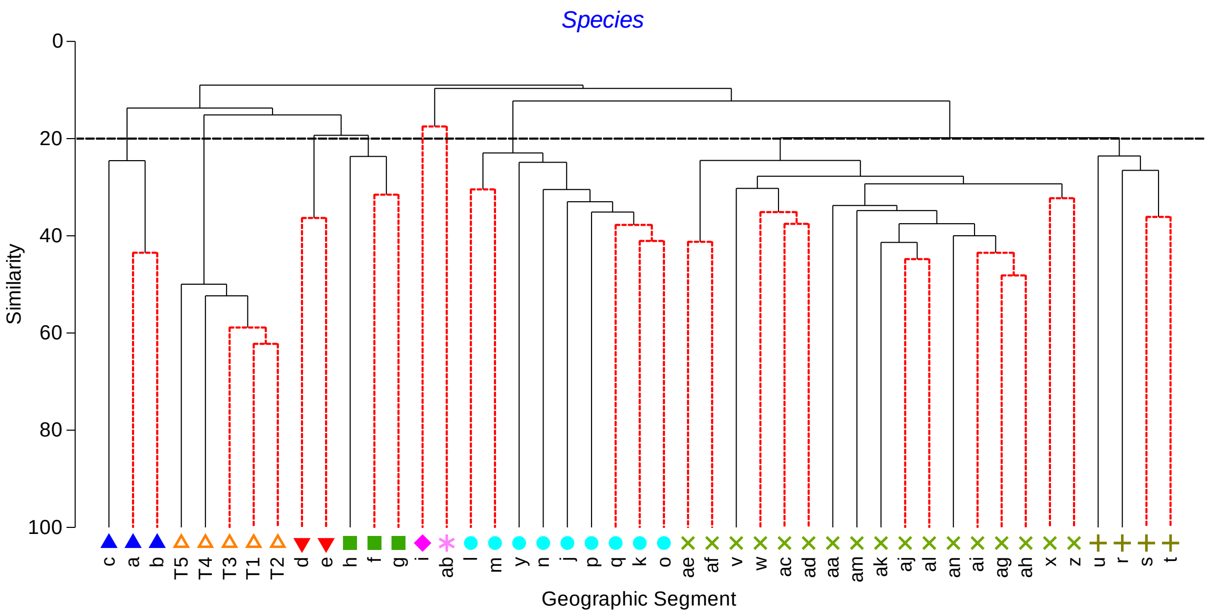
Species
Similarity
0
20
40
60
80
100
c a b T5 T4 T3 T1 T2 d e h f g i ab l m y n j p q k o ae af v w ac ad aa am ak aj al an ai ag ah x z u r s t
Geographic Segment

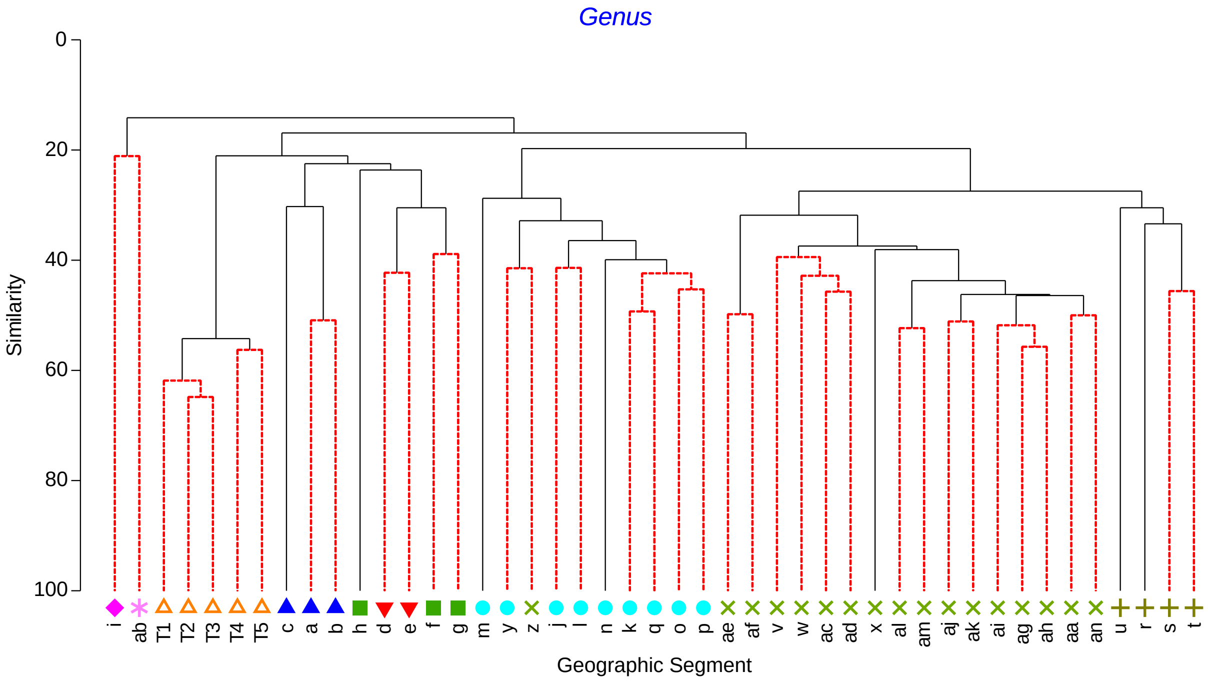
Genus
Similarity
0
20
40
60
80
100
i ab T1 T2 T3 T4 T5 c a b h d e f g m y z j l n k q o p ae af v w ac ad x al am aj ak ai ag ah aa an u r s t
Geographic Segment

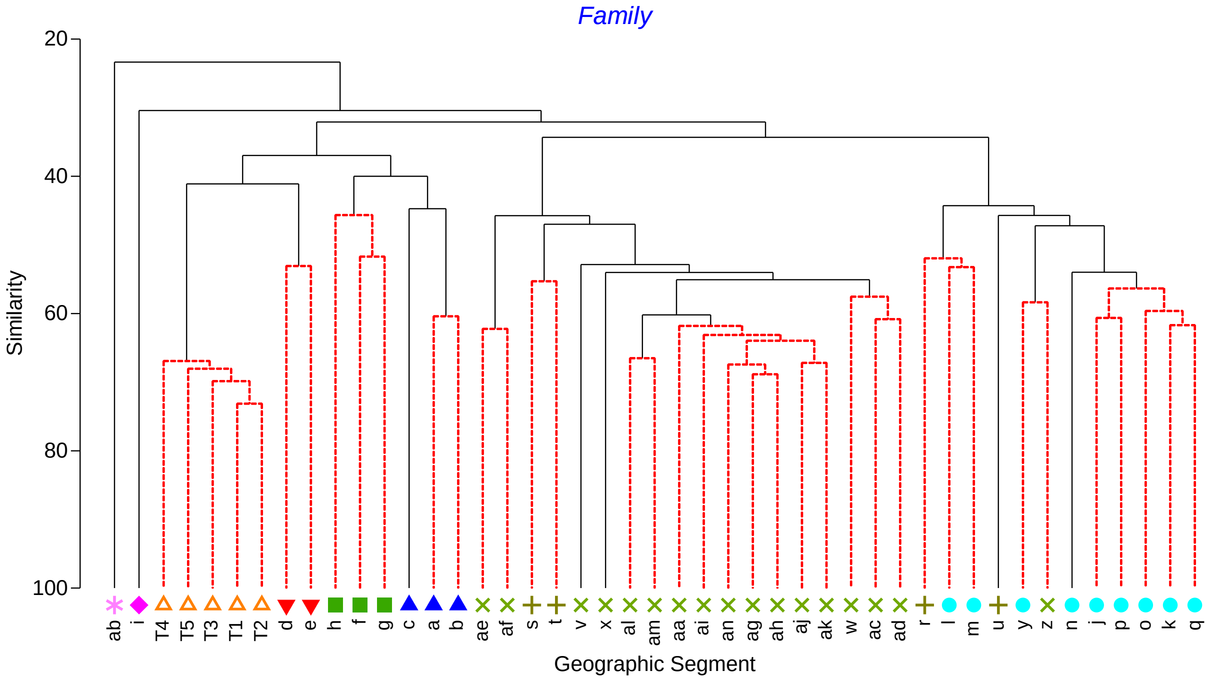
Family
Similarity
20
40
60
80
100
ab i T4 T5 T3 T1 T2 d e h f g c a b ae af s t v x al am aa ai an ag ah aj ak w ac ad r l m u y z n j p o k q
Geographic Segment

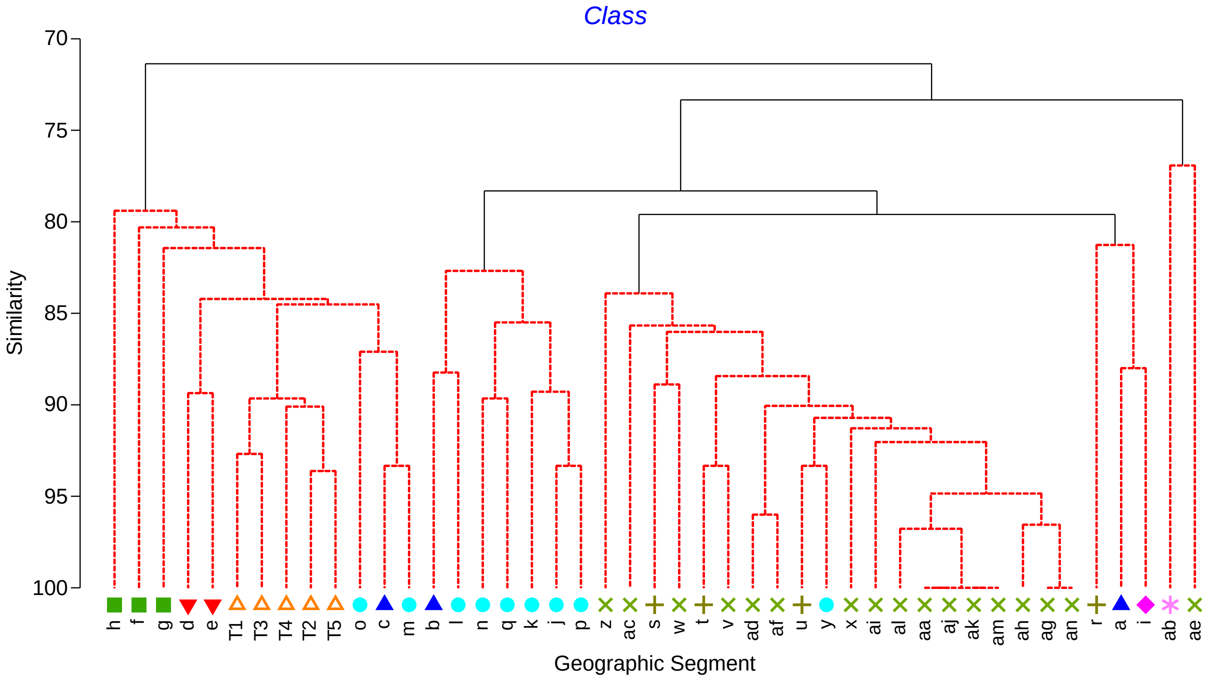
Class
Similarity
70
75
80
85
90
95
100
h f g d e T1 T3 T4 T2 T5 o c m b l n q k j p z ac s w t v ad af u y x ai al aa aj ak am ah ag an r a i ab ae
Geographic Segment

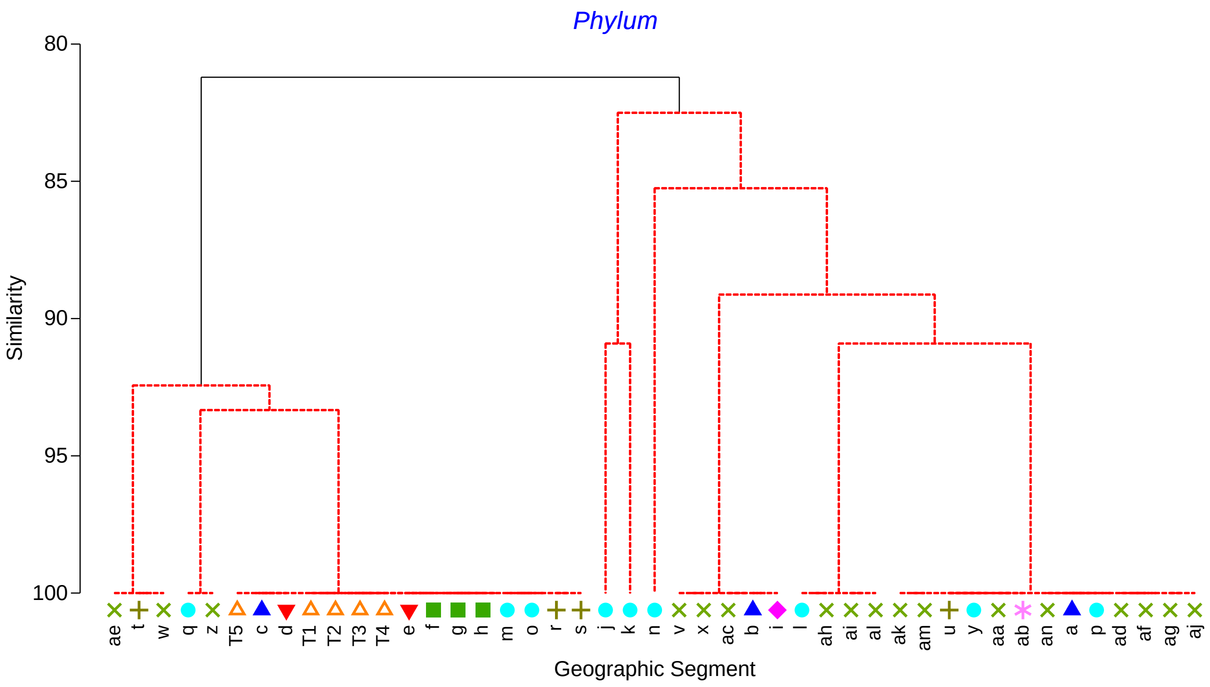

Supplementary Figure 2 Cluster analysis of southern Australian deep-sea benthos at different taxonomic levels (see Figure 1 for geographic locations of each point). Red lines indicate groupings that do not differ at the 5% significance level. Colour coding indicates 20% similarity level from the species level cluster analysis. Note that the species-level plot is the same as in Figure 3, and is presented again for ease of comparison.
